# Supplementary material for: Clinical Characteristics of Obstructive Sleep Apnea in Psychiatric Disease
Source: J Clin Med. 2019 Apr 18;8(4):534. doi: 10.3390/jcm8040534 (PMC6518048; doi:10.3390/jcm8040534)
Supplement: Supplementary file 1 [file jcm-08-00534-s001.pdf]

Table S1. The distribution of the self-reported symptoms and the demographic items in the STOP-Bang Sleep Apnea questionnaire.

STOP-Bang Questionnaire: Self-Reported Symptoms	Yes	No
Do you snore loudly (louder than talking or loud enough to be heard through closed doors)?	76.42	23.58
Do you often feel tired, fatigued, or sleepy during daytime?	68.87	31.13
Has anyone observed you stop breathing during your sleep?	59.05	40.95
Do you have or are you being treated for high blood pressure?	37.74	62.26
**STOP-Bang Questionnaire: Demographic Items**	**Yes**	**No**
BMI > 35 Kg/m^2?	32.14	67.86
Age > 50 years old?	65.18	34.82
Neck circumference > 40 cm	49.5	50.5
Gender: male?	49.11	50.89
